# What’s Next for Flu? Out-of-Season Circulation of Influenza Viruses in Southern Italy, August 2022

**DOI:** 10.3390/v14122689

**Published:** 2022-11-30

**Authors:** Daniela Loconsole, Francesca Centrone, Valerio Aprile, Anna Sallustio, Daniele Casulli, Marisa Accogli, Davide Sacco, Riccardo Zagaria, Maria Chironna

**Affiliations:** 1 Department of Interdisciplinary Medicine, Hygiene Section, University of Bari, 70124 Bari, Italy; 2 Department of Prevention, Local Health Unit of Lecce, 73100 Lecce, Italy; 3 Hygiene Unit, Azienda Ospedaliero-Universitaria Consorziale Policlinico di Bari, 70124 Bari, Italy

**Keywords:** influenza A virus, out-of-season circulation, outbreak, respiratory viruses, epidemiology, phylogenetic analysis, SARS-CoV-2

## Abstract

The COVID-19 pandemic has modified the seasonal pattern of respiratory infections. The objective of the present study is to characterize the out-of-season circulation of influenza viruses and an influenza outbreak that occurred in southern Italy in August 2022. Nasopharyngeal swabs collected from patients with influenza-like illnesses (ILI) were tested for the presence of influenza and other respiratory viruses. Epidemiological investigations on 85 patients involved in an influenza outbreak were performed. Sequencing and phylogenetic analysis of hemagglutinin genes was undertaken on samples positive for influenza A. In August 2022, in the Apulia region (Italy), influenza A infection was diagnosed in 19 patients, 18 infected with A/H3N2 and one with A/H1N1pdm09 virus. Seven influenza-positive patients were hospitalized with ILI. A further 17 symptomatic subjects, associated with an influenza outbreak, were also tested; 11 were positive for influenza A/H3N2 virus. Phylogenetic analysis of 12 of the A/H3N2 sequences showed that they all belonged to subclade 3C.2a1b.2a.2. The A/H1N1pdm09 strain belonged to subclade 6B.1A.5a.2. The out-of-season circulation of the influenza virus during the summer months could be linked to changing dynamics in the post-COVID-19 era, as well as to the impact of climate change. Year-round surveillance of respiratory viruses is needed to monitor this phenomenon and to provide effective prevention strategies.

## 1. Introduction

Influenza is a major, worldwide public health concern. Prior to the 2009 pandemic, influenza was considered a mild, seasonal disease involving older age groups. The 2009 pandemic was sustained by circulation of the influenza A/H1N1pdm09 virus and was less severe than previous pandemics; indeed, it quickly evolved into a seasonal transmission pattern [1]. Influenza epidemics account for an estimated 1 billion annual cases worldwide, of which 3–5 million result in severe disease. Older adults, in particular, show a high burden of severe cases, with a high rate of hospitalizations and deaths [2]. Moreover, an estimated 290,000–650,000 influenza-related deaths occur every year [3,4].

Seasonality patterns of respiratory viruses are well-established and depend on geographic areas and climate conditions. In temperate regions, influenza viruses circulate mainly in the winter months, while in tropical and subtropical areas, influenza epidemics may occur throughout the year, with irregular outbreaks [5]. In Italy, influenza places a significant burden across all age groups every year, particularly among the elderly, children, and people with comorbidities, with a relevant excess in mortality rate [6]. In the pre-pandemic era, the peak of incidence of influenza cases was usually reported between January and February [7]. It has been reported that, in the general population, complications related to influenza occurred in 35% of patients requiring general practitioner visits, with the elderly and people with underlying chronic conditions representing the population with significantly increased risk [6]. Bronchitis and pneumonia are the most common complications and the most reported causes of hospitalization. In particular, hospitalization has been reported in 0.43% of the general population, and 0.7% of the pediatric population [6].

Changes in the behavioral habits of the population, induced by the COVID-19 pandemic, have modified the epidemiology of common respiratory infections [8,9]. In particular, a global reduction in influenza circulation has been observed, with the decline in circulation attributed to non-pharmaceutical interventions (NPIs) implemented to limit the spread of SARS-CoV-2 [10], and to viral competition resulting from the widespread circulation of the SARS-CoV-2 virus [11,12]. In many countries, the seasonal activity of influenza viruses has been massively suppressed [13,14]. The lower rate of infections registered in 2021 could also be a consequence of the higher influenza vaccination coverage achieved in that year than in the years before the COVID-19 pandemic [15]. 

The 2021–2022 influenza season in the Northern Hemisphere was characterized by a biphasic trend, with A/H3N2 viruses being the dominant strain in all countries [16]. In the United States, the first peak of the season occurred in Week 51/2021 and the second peak in Week 16/2022 [17]. In Europe, influenza activity first peaked in Week 52/2021, declining thereafter until Week 4/2022, whereupon it increased again, reaching the plateau phase between Weeks 10/2022 and 15/2022 [16]. Similarly, in Italy, the number of influenza cases reached a first peak in Week 52/2021 (incidence: 5.16/1000 population), with a second peak in Week 13/2022 (incidence: 5.29/1000 population) [18]. The second increase in influenza cases seen in the Northern Hemisphere represents a later activity than those seen in most previous seasons [16]. The objective of the present study was to describe a worrisome out-of-season circulation of influenza viruses, and an outbreak that occurred in southern Italy in August 2022.

## 2. Materials and Methods

### 2.1. Subject Enrollment

For the purpose of the study, data and samples collected through the routine influenza surveillance of the Apulia region were used. Moreover, cases identified through the epidemiological investigation of an outbreak that occurred in 85 subjects after attending a religious retreat and residing in the Apulia region were also used. In Italy, the regional influenza surveillance system surveys about 4% of the population, as recommended by the national protocol [19]. In the Apulia region (population, >4 million people), epidemiological and virological surveillance is managed by the Regional Observatory for Epidemiology, as previously described [20]. For the season relating to this study, seasonal virological surveillance was scheduled to start from Week 46/2021 and end in Week 17/2022. However, due to the prolonged circulation of influenza viruses observed in 2022, virological surveillance continued after Week 17/2002. To better explain the usual seasonal circulation pattern of influenza viruses in the Apulia region, all the influenza-positive samples collected by the regional reference laboratory for surveillance purposes from season 2015–2016 to season 2021–2022 were considered for the construction of the epidemic curves.

### 2.2. Molecular Identification and Sequencing of Influenza Viruses

Samples were analyzed at the Laboratory of Molecular Epidemiology and Public Health of the Hygiene Unit of Policlinico Hospital of Bari, which is the regional reference laboratory for the virological surveillance of influenza. Viral nucleic acids were extracted using the STARMag Universal Cartridge Kit (Seegene, Seoul, Korea) on the automated Nimbus IV platform. Real-time PCR was performed using the AllplexTM respiratory panel assays (Seegene, Seoul, Korea) to detect 16 different viruses, including the influenza A and B viruses, RSV A and B, adenovirus, enterovirus, parainfluenza viruses 1–4, metapneumovirus, bocavirus, rhinovirus, and three coronaviruses (NL63, 229E, and OC43). Samples were also analyzed using AllplexTM SARS-CoV-2 assays (Seegene, Seoul, Korea) to detect SARS-CoV-2. Sequencing of the hemagglutinin (HA) gene was performed using conventional Sanger sequencing. The SuperScript III One-Step PCR Amplification Kit (Invitrogen, Monza, Italy) was used for HA gene amplification according to the WHO protocol [21], and PCR products were purified using a PCR purification kit (Qiagen, Milano, Italy). Sequences were assembled and analyzed by CLC DNA Workbench 6.0 (Qiagen). Molecular Evolutionary Genetics Analysis (Mega) 11 software (https://www.megasoftware.net/, accessed on 30 November 2022) was used to align sequences by the Clustal W method, to compare the sequences with those contained in the GenBank and Global Initiative on Sharing Avian Influenza Data (GISAID) databases, and to generate phylogenetic trees. Amino acid substitutions were identified by comparing the HA gene sequences of the identified strains and the HA gene sequences of the reference strains through the Nextclade tool (https://clades.nextstrain.org/, accessed on 30 November 2022). All the sequences generated in this study were deposited in the GISAID database. Sequence accession numbers are reported in the phylogenetic tree. 

### 2.3. Ethical Statement

Epidemiological investigations of cases involved in the outbreak were performed by the Department of Prevention of the Local Health Unit of Lecce (Apulia, Italy).

The activities described in this study were part of the legislated mandate of the Health Promotion and Public Health Department of the Apulia region (Italy); therefore, approval of this study by the ethics committee was waived. All procedures were performed in accordance with the Declaration of Helsinki, as revised in 2013, for research involving human subjects. 

## 3. Results

**Figure 1:**
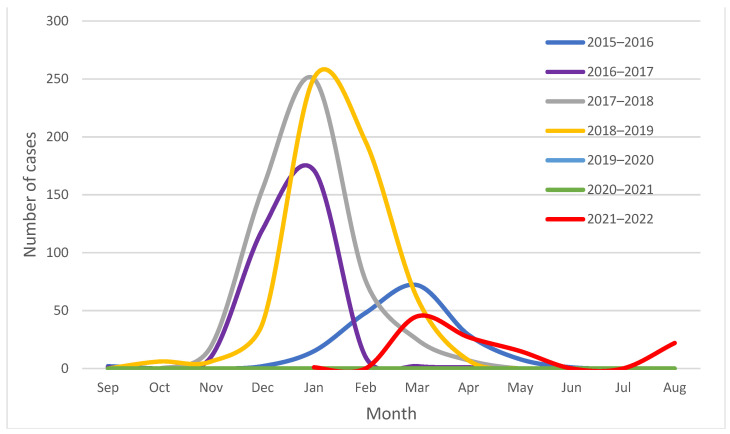
Seasonal circulation pattern of influenza viruses in the Apulia region of Italy, from season 2015–2016 to season 2021–2022.

Between 1 September 2015 and 31 August 2022, 1700 influenza-positive samples were collected for surveillance purposes in the Apulia region of Italy. Based on these data, the epidemic curves showing the usual seasonal circulation pattern of influenza viruses in the Apulia region from season 2015–2016 to season 2021–2022 have been constructed (Figure 1).

Between 14 and 31 August 2022, 19 cases positive for influenza A virus were identified in the Apulia region. On 14 and 16 August 2022, two sporadic A/H3N2 influenza cases were identified in children (aged 2 and 15 years, respectively) hospitalized with an influenza-like illness. The two children were from different provinces and neither had any history of travel out of the region in the weeks prior to their hospitalization. 

Around the same time, an outbreak of influenza A virus was identified in a group of 85 adolescents and adult guides residing in the south of Apulia, after attending a religious retreat in a sanctuary in the Basilicata region (southern Italy). The clinical and demographic characteristics of the patients involved in the outbreak related to the religious retreat are reported in Table 1. 

Four participants had resided in the venue from 7–17 August 2022 (group A), 73 participants from 8–14 August 2022 (group B), and eight participants from 12–17 August 2022 (group C). Nasopharyngeal swabs were taken from 17 symptomatic patients (20%), all from group B. Of these, nine were positive for influenza A/H3N2 virus, two were positive for influenza A/H3N2 and rhinovirus, two were positive for rhinovirus, and four were negative for all the tested viruses. 

All the influenza-positive subjects who showed symptom onset between 8 and 11 August 2022 belonged to group B. Overall, the average length between the onset of symptoms and the date of sample collection was 5 days (range: 3–10 days), while for negative subjects it was 7 days (range: 6–9 days). All symptomatic patients tested negative for SARS-CoV-2.

Between 22 and 31 August 2022, five further cases of influenza A/H3N2 virus (with no linkage to one another) and one case of A/H1N1pdm09 infection were also identified. One A/H3N2 case was in an adult subject returning from Ibiza (Spain), and the other four A/H3N2 cases were all in hospitalized children. The A/H1N1pdm09 case was in an adolescent with no history of travel in the previous weeks. 

**Figure 2:**
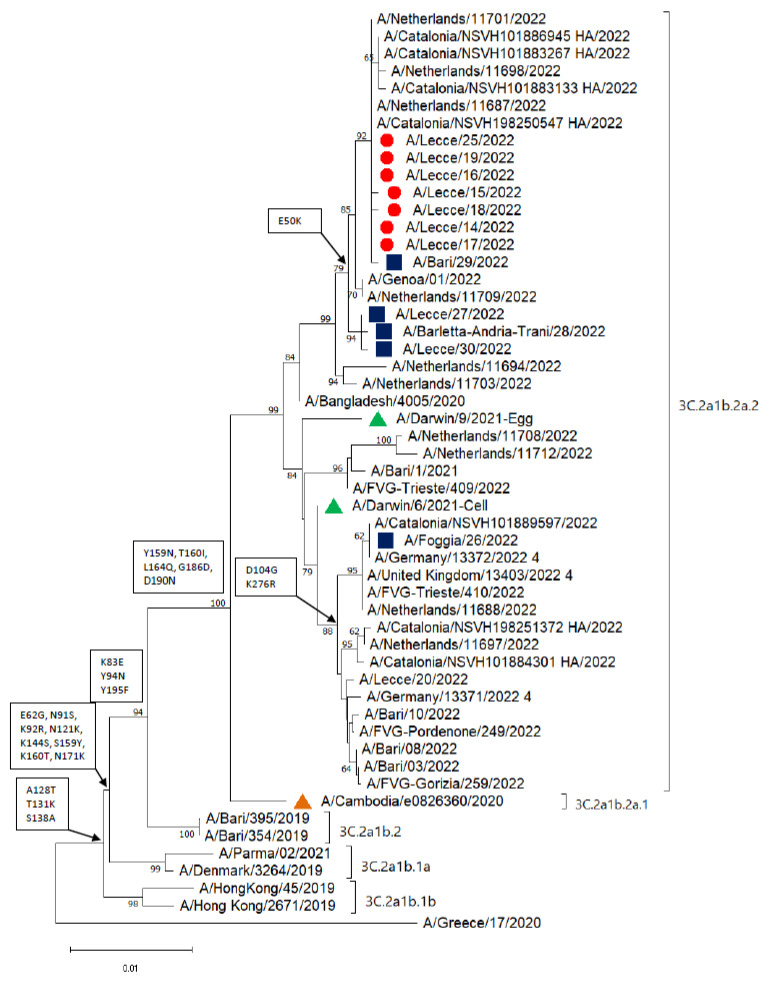
Phylogenetic analysis of the hemagglutinin (HA) gene of 12 A/H3N2 influenza viruses characterized in the Apulia region of Italy in August 2022. Red dots represent strains identified from the outbreak and blue squares represent sporadic strains; green triangles represent the vaccine strains for the 2022–2023 season, and the orange triangle represents the vaccine strain for the 2021–2022 season. The phylogenetic tree was constructed using Mega 11 software, applying the neighbor-joining algorithm. The evolutionary distances were calculated using the Kimura 2-parameter method. Amino acid substitutions defining specific genetic clusters are shown at the nodes. Genetic clades are shown to the right of the tree, and the scale bar represents the nucleotide substitutions per site (GISAID accession numbers: EPI2155546, EPI2155545, EPI2155544, EPI2155543, EPI2155541, EPI2155535, EPI2155534, EPI2155533, EPI2155532, EPI2163106, EPI2179877, and EPI2179878).

Phylogenetic analysis was performed on the HA gene of 12 A/H3N2 sequences, seven of which were associated with the outbreak (Figure 2). 

All the sequences clustered into subclade 3C.2a1b.2a.2 (reference strains A/Bangladesh/4005/2020 and A/Darwin/9/2021; the latter is the vaccine strain for the Northern Hemisphere for the 2022–2023 season). All the strains from the outbreak and the sporadic cases, except for the A/Foggia/26/2022, clustered with strains isolated in Spain and the Netherlands in August 2022, and with an Italian strain identified in a sample collected on 22 August 2022 (A/Genoa/01/2022). The strain A/Foggia/26/2022, isolated from the patient returning from Spain, clustered separately with strain A/Catalonia/NSVH101889597/2022. The HA gene of all the strains identified in the Apulia region during the summer of 2022 encoded Y159N, T160I, L164Q, G186D, and D190N amino acid substitutions when compared with the HA gene sequence of the A/Cambodia/e0826360/2020 reference strain. Moreover, the HA genes of all the strains isolated from the outbreak, along with those from four of the sporadic cases, encoded an E50K substitution, while the A/Foggia/26/2022 strain also encoded D104G and K267R substitutions (Figure 2).

The single A/H1N1pdm09 strain (accession number: EPI2176758) belonged to subclade 6B.1A.5a.2, which is the same subclade as the 2022–2023 season vaccine strains (A/Victoria/2570/2019 and A/Wisconsin/588/2019).

## 4. Discussion

An out-of-season circulation of influenza viruses, which was associated with an outbreak of A/H3N2 influenza virus, was identified in the Apulia region of Italy during August 2022. Summer season epidemics of influenza are common in subtropical areas, where they are the principal cause of acute respiratory infections in children and adults [22]. All of the influenza cases in this study were caused by influenza A viruses, whereas in other countries such as Japan, the influenza B virus has been the most frequent cause of summer epidemics [22]. In Italy, the influenza B virus has not been in circulation since the 2017–2018 influenza season [20,23]. It could be hypothesized that both the geographical area and the different climate could have influenced the spread of virus A at our latitudes and also in the summer season. 

Nineteen subjects involved in the outbreak connected to the religious retreat showed symptoms between 8 and 11 August 2022, all of them residing in the location of the religious retreat from 8–14 August 2022 (group B). Since the incubation period of influenza ranges from 1–4 days, with an average of 2 days [1], we can speculate that these individuals acquired the infection before arriving at the retreat. This hypothesis is also supported by phylogenetic analysis, which showed that the sequences of the A/H3N2 viruses recovered from the retreat patients were closely related to the sequences of influenza A viruses found in other symptomatic patients, with no apparent epidemiological linkage, in the Apulia region.

The influenza virus-negative results for six patients associated with the outbreak could be explained by the time taken for sample collection after symptom onset (average: 7 days). 

Influenza viruses circulating in the summer months usually diverge from the previous winter viruses, and more closely resemble the viruses detected in the following season [22]. The influenza A/H3N2 viruses detected in this study are all closely related to the 2022–2023 season vaccine strains (A/Darwin/6/2021 and A/Darwin/9/2021). Similarly, the single A/H1N1pdm09 strain detected is closely related to the A/H1N1pdm09 strains included in the seasonal vaccine.

The circulation of influenza viruses in the summer months in temperate regions could be considered a sentinel event during changes in the epidemiology of respiratory viruses after the COVID-19 pandemic. Due to the rapid waning of immunity after influenza vaccination, the availability of year-round influenza vaccines, even during the out-of-season circulation period, could prevent a high burden of disease [24]. Based on the recommendations of the Advisory Committee on Immunization Practices, influenza vaccines for the 2022–2023 influenza season might be available as early as July or August. However, vaccination during these months is not recommended for adults aged ≥65 years, or for pregnant women in the first or second trimester, because of the possible waning of immunity over the course of the influenza season [25]. The vaccine coverage recorded in the Apulia region in the 2021–2022 influenza season was lower than in the previous year [26]. However, even with high vaccine coverage, the population may not have been fully protected against influenza viruses since protective antibodies induced by immunization are efficacious for only a few months after administration of the vaccine, and not for the whole year [1,24]. In previous seasons, low vaccine efficacy against A/H3N2 was demonstrated in Italy [27]. In the cases described here, a mismatch between the circulating influenza A/H3N2 virus and the 2021–2022 vaccine strain (A/Cambodia/e0826360/2020) was identified. Moreover, since almost all of these cases related to young people, high vaccination coverage would not have impacted this out-of-season circulation of influenza viruses. 

Based on the occurrence of out-of-season outbreaks of respiratory viruses such as influenza [24,28] and respiratory syncytial viruses [29,30], other unexpected outbreaks might occur in periods of COVID-19 remission. At the time of writing, further influenza A/H3N2 viruses have been identified in hospitalized patients, particularly children, in the Apulia region, indicating long-lasting circulation of this virus, as well as community spread of the infection, in an out-of-season period. The implementation of NPIs to mitigate the spread of SARS-CoV-2 impacted the circulation of influenza viruses worldwide [10]. The dynamics of novel respiratory diseases in the post-pandemic era could be driven by discontinuation of the mitigating measures, but also by the presence of other prevailing competing viruses in the ecosystem, as well as by changing weather patterns [24,31]. In Brazil, the occurrence of an unexpected diffusion of influenza virus A/H3N2 in the late spring of 2021 was linked to the lower-than-expected mean temperature registered in that period [24]. It is well-known that confinement of people to indoor settings increases person-to-person contact and facilitates virus transmission [32]. Therefore, we could speculate that environmental factors could have also promoted the out-of-season circulation of influenza viruses described here. Further studies would clarify the role of climate change in the changing epidemiology of respiratory viruses. 

This study has some limitations. First, the whole-genome sequencing of the identified strains was not performed. However, sequencing the HA gene is the standard method for the molecular characterization of influenza viruses. Moreover, the results of the molecular characterization of the HA gene allowed a rapid national sharing of data to alert the community about this out-of-season circulation of influenza viruses. Second, only 20% of the subjects involved in the outbreak were tested through a nasopharyngeal swab. However, the epidemiological investigation was sufficient to link all the cases. 

The summer circulation of seasonal influenza viruses here described has been considered an epidemic alert since influenza viruses had never been detected during the summer months in Italy, except for the 2009 pandemic flu. Based on the out-of-season circulation of respiratory viruses, a sensitive, nationwide, year-round surveillance system of SARS-CoV-2, influenza, and respiratory syncytial virus should be set up for monitoring the circulation of such viruses [33]. This is particularly the case when other, new, or more virulent viruses emerge since, like SARS-Cov-2, they may also change the dynamics of the viruses under surveillance, triggering significant outbreaks at atypical times [22]. Moreover, gaining insight into the circulation of other respiratory viruses is of utmost importance to guide decisions on preventive strategies in public health. A particular concern is that the reopening of schools in the next fall season will result in more widespread influenza outbreaks, and a more severe influenza season, since fewer children were naturally exposed to influenza viruses during the COVID-19 pandemic period [34].

## 5. Conclusions

Seasonal patterns of respiratory viruses have been unpredictable during the COVID-19 pandemic. These changing dynamics could be driven by reduced pathogen exposure that is due to the robust implementation of NPIs, as well as to the impact of climate change. Effective surveillance is a crucial point of pandemic influenza preparedness since it allows close monitoring of circulating viruses and the early detection of, and responses to, any unusual activity that could result in large-scale outbreaks.

## Figures and Tables

**Table 1:** Clinical and demographic characteristics of patients involved in an A/H3N2 influenza outbreak related to the religious retreat, in the Apulia region of Italy, in August 2022.

	N	%
Patients	85	100%
Sex		
Female	45	52.9%
Male	40	47.1%
Median age (IQR ^1^)	13 (10–21)	
Clinical status		
Asymptomatic	20	23.5%
Symptomatic	65	76.5%
Fever	45	52.9%
Cough and/or sore throat	53	62.4%
Cold	15	17.6%
Headache	13	15.3%
Otalgia and/or otitis	3	3.5%
Nausea and/or vomiting	17	20.0%
Diarrhea and/or abdominal cramps	2	29.4%
Dyspnea	1	1.2%
Neurological symptoms (stunning, confusion)	3	3.5%
General malaise	33	38.8%
Tested nasopharyngeal swab	17	20.0%
Positive for influenza A/H3N2	11	12.9%
Positive for SARS-CoV-2	0	
Positive for rhinovirus	4	4.7%
Negative ^2^	4	4.7%
Flu vaccination (2021/2022)	14	16.5%
Travel in the last 2 weeks	19	22.4%

^1^ IQR = interquartile range; ^2^ negative for all the tested viruses.

## Data Availability

The data that support the findings of this study are available on request from the corresponding authors. The data are not publicly available because of privacy or ethical restrictions.
